# Fetal Congenital Diaphragmatic Hernia and Hydramnios in a Quarter Horse Mare

**DOI:** 10.3390/vetsci8100201

**Published:** 2021-09-22

**Authors:** Aliai Lanci, Martina Ingallinesi, Maria Morini, Francesca Freccero, Carolina Castagnetti, Jole Mariella

**Affiliations:** 1 Department of Veterinary Medical Sciences, University of Bologna, Via Tolara di Sora 50, Ozzano dell’Emilia, 40064 Bologna, Italy; martina.ingallinesi@unibo.it (M.I.); maria.morini@unibo.it (M.M.); francesca.freccero2@unibo.it (F.F.); carolina.castagnetti@unibo.it (C.C.); jole.mariella2@unibo.it (J.M.); 2 Health Science and Technologies Interdepartmental Center for Industrial Research (HST-ICIR), University of Bologna, Via Tolara di Sopra 41/E, Ozzano dell’Emilia, 40064 Bologna, Italy

**Keywords:** hydramnios, congenital diaphragmatic hernia (CHD), prepubic tendon rupture, equine, foal, mare, hydrops

## Abstract

Hydramnios is an excessive accumulation of fluid within the amniotic compartment. It is a rare condition in mares, often associated with fetal anomalies. Hydrops of fetal membranes predisposes to the rupture of the prepubic tendon, and many authors suggest the induction of parturition to preserve mare’s reproductive career. This report presents the case of a 15-year-old multiparous Quarter Horse mare, referred at 268 days of gestation for suspected hydrops. Repeated ultrasonographic exams confirmed an increase in the depth of the amniotic fluid and reduced fetal viability. During the hospitalization, the mare developed a partial rupture of the prepubic tendon. In this case, a conservative approach was elected, and the mare was treated with nonsteroidal anti-inflammatory drugs (NSAIDs) and an abdominal support bandage. At 327 days of gestation, the mare gave birth to a foal with APGAR score 1. The resuscitation attempt was unsuccessful, and the foal died immediately. A post-mortem examination diagnosed a congenital diaphragmatic hernia (CDH) with pleuroperitoneal diaphragmatic eventration.

## 1. Introduction

In mares, hydrallantoid and hydramnios are rare conditions, characterized by the pathological accumulation of fluid within the allantoic and amniotic compartments, respectively. Only a few cases of hydramnios in mares are described in the literature [1,2,3], and only one resulted in the birth of a live foal [4]. In the amniotic compartment, normal fetal fluid volume is reported to range from about 3 to 5 L near term; in the allantoic compartment, there is a gradual increase to about 8–15 L [5]. In the case of hydrops, the amount of accumulated fluid is variable: an increase in allantoic fluid to 120–220 L was reported by Vandeplassche et al. [6], whereas Honnas et al. [1] described a 96 L hydramnios.

In cows, the pathophysiology of hydrallantoid has been related to abnormal placentation, whereas hydramnios has been associated with an abnormality of the fetal head that precludes swallowing [7]. In one mare, the presence of fetal anomalies in the head, and in particular, in the oral cavity in the case of hydramnios, would support this thesis [2]. Conversely, the experimental induction of esophageal atresia in four ovine fetuses during advanced gestation did not result in hydramnios, suggesting the existence of other mechanisms in the pathogenesis of this condition [8].

Frequent complications of hydrops are the rupture of the prepubic tendon [9] and body wall tears [10]; in most serious cases, rupture of the uterus is also reported [1].

The present case report describes a case of hydramnios in a mare, resulting in the full-term birth of a non-viable foal with congenital abnormality.

## 2. Clinical Description

In March 2021, a 15-year-old multiparous Quarter Horse mare at 268 days of gestation was referred to the Perinatology and Reproduction Unit (Equine Clinical Service, Department of Veterinary Medical Sciences) of the University of Bologna for premature udder development and suspected hydrops. The referring veterinarian had noticed an increase in the volume of amniotic fluid during a transrectal ultrasound examination.

**Figure 1:**
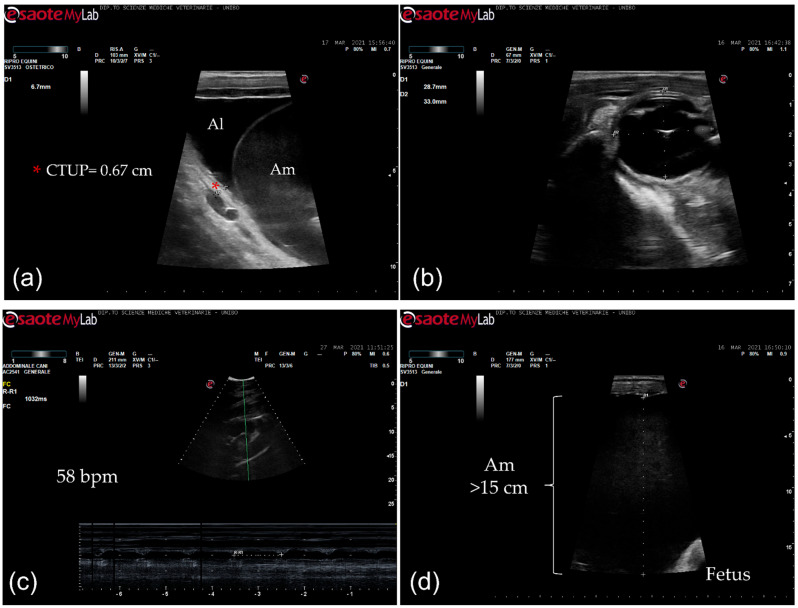
Ultrasonographic images. (**a**) Transrectal ultrasonographic examination of the combined thickness of the uterus and placenta (CTUP) (red asterisk); Al: allantoic fluid; Am: amniotic fluid; (**b**) fetal orbit measurement (transrectal ultrasonography); (**c**) fetal heart rate measurement (58 bpm) (transabdominal ultrasonography); (**d**) depth of the amniotic fluid (>15 cm) (transabdominal ultrasonography); Am: amniotic fluid.

The mare had never shown any problems in the previous seven pregnancies. At admission, the mare presented with severe edema of the ventral portion of the abdomen and caudally to the udder. The mare was reluctant to move and sweaty. Body condition score and muscular tone were normal. At physical examination, she presented tachycardia (65 beats/min) and slight tachypnea (20 breaths/min), and the body temperature (37.6 °C) was within normal limits. On abdominal auscultation, the motility was decreased, and fecal output was reduced, with dry feces throughout hospitalization. The mammary gland was well developed. Mammary secretions were evaluated with a calcium titration method (FoalWatch; Chemetrics, Midland, VA, USA) and the concentration of calcium was 150 ppm Ca [11]. Hemato-chemistry parameters were within normal limits and the serum biochemistry was monitored during the hospitalization period. Transrectal ultrasonographic (US) examination showed a combined thickness of the utero-placental unit (CTUP) within normal limits (range 5.53–6.77 mm at 10 months of pregnancy) (Figure 1a) [12]. The fetus was in anterior presentation, and the orbital size was 33 × 28.7 mm (Figure 1b). Transabdominal ultrasonographic examination showed a fetal heart rate at rest at the lower limit (58 bpm) (Figure 1c) and lower than the normal range after activity (67–71 bpm) [13,14]. The maximum depth of the amniotic fluid was measured in the mid-caudal quadrant (>15 cm; Figure 1d); in the mid–mid quadrant, the allantoic fluid was measured at the maximal depth (11 cm) [14].

On the third day of hospitalization, the mammary secretions were bloody. For this reason, a partial rupture of the prepubic tendon was suspected, supported by the increase in serum creatine kinase (CK) enzyme activity (Table 1). The mare was treated with flunixin meglumine (1.1 mg/kg i.v. q12h) and sucralfate (20 mg/kg per os q8h) and kept on stall rest. A commercial abdominal support bandage was applied, and the wrap was tightened daily as the ventral edema and swelling decreased over time. On day 316 of gestation, NSAID therapy was changed to firocoxib (loading dose 0.3 mg/kg per os SID, then 0.1 mg/kg per os SID) [15].

Further ultrasonographic examination (US) checks were performed every 5 days, until delivery. Repeated US exams confirmed a persistent increase in the depth of the amniotic fluid compared to the reference values [13,14], reduced fetal viability [14,16], and reduced fetal movements. The fetal stomach was never visualized.

In the following days, mammary secretions became milky, and mammary calcium was 250 ppm; moreover, the mare began to drop secretions.

On day 321 of gestation, the maximal abdominal circumference was 230 cm; six days later, it became 320 cm.

**Figure 2:**
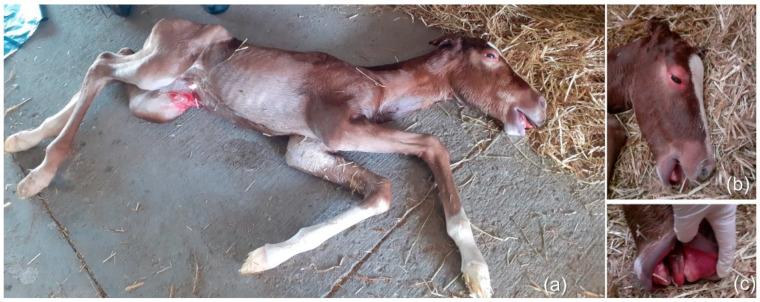
Macroscopic findings of the foal (**a**) foal at birth; (**b**) edema of the head, eyelids and lips; (**c**) congested mucous membranes.

At 327 days of gestation, the mare gave birth to a non-viable male foal (Figure 2a). The foaling took place in broad daylight, and it lasted about 20 min. The foal was born at 3:20 pm still wrapped inside the amniotic membrane, which required a certain force to be broken. The APGAR score at birth was 1: heart rate was 40 bpm; mucous membranes were congested/cyanotic (Figure 2c); the foal was unresponsive to stimulation, in lateral recumbency, and spontaneous respiration did not commence. The resuscitation attempt was unsuccessful, and the foal died immediately. The head was edematous, particularly at the level of the lips and eyelids (Figure 2b).

Foal’s blood lactate was measured after death by intracardiac blood sampling and measured at 17 mmol/L; biochemical parameters and fibrinogen were also evaluated (Table 2). The mare’s blood lactate after parturition was 1.1 mmol/L and blood glucose 115 mg/dL, both measured from jugular blood.

**Figure 3:**
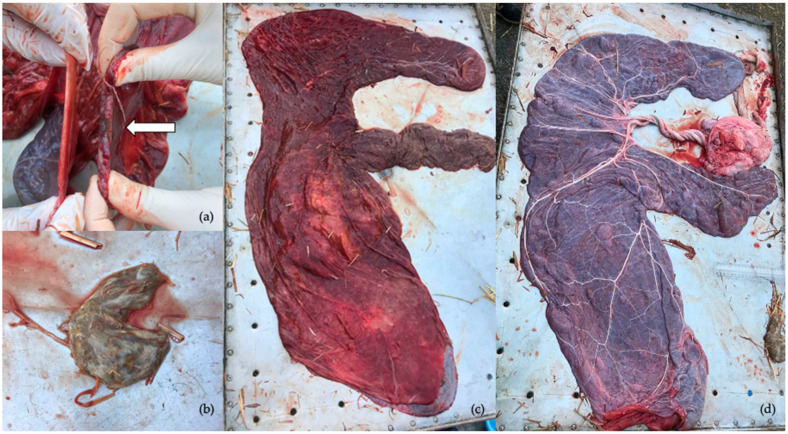
Gross examination of the fetal membranes (**a**) Thickening of a portion of the cervical star; (**b**) hippomane; (**c**) chorionic surface of fetal membranes; (**d**) allantoic surface.

The fetal membranes were expelled after 50 min from parturition. Their total weight was 4.3 kg (chorionallantois 3.25 kg). The length of the cord in the amniotic portion measured 42 cm with five coils, whereas in the allantoic portion, it was 35 cm with four coils. On the chorionic surface, the cervical pole presented an edematous portion 1–1.2 cm in thickness, whereas another portion was very thin (Figure 3a). On the body, there was an edematous portion about 2 cm thick, but the rest was normal. The villi distribution was homogeneous, with a slight rarefaction in the body within normal limits (Figure 3c). The attachment of the umbilical cord was between the two horns and the pregnant horn was the left one. The allantoic surface was normal (Figure 3d). It was possible to appreciate a thickening of some portion of the amniotic membrane. An anomalous hippomane was detected, with heterogeneous color and soft consistency (Figure 3c). Full-thickness samples of the chorioallantois were taken from the cervical star, the body, the non-gravid horn and the gravid horn [18,19]. Samples were formalin-fixed and paraffin-embedded. Routine histological hematoxylin–eosin-stained slides were obtained. The histopathological exam of the chorioallantois showed diffuse edema of the chorionic connective lamina.

**Figure 4:**
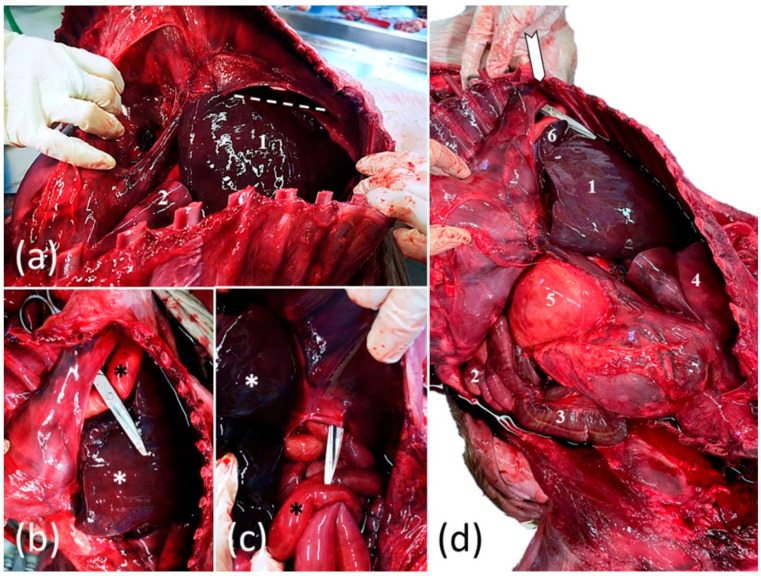
Foal’s congenital pleuroperitoneal diaphragmatic hernia: (**a**) cranial view of the thoracic cavity after removal of the left thoracic wall; the thoracically translocated parts of the liver (1), the diaphragm and the area of the dorsal diaphragmatic defect (dotted accolade) and hypoplastic left lung (2); (**b**,**c**) caudal view of the large diaphragmatic defect with round and bold margins (indicated by surgical scissors) which allowed partial migration of the liver (white *) and bowel (black *) from the abdomen into the thorax; (**d**) lateral view of the coelomic cavity after removal of the thoracic wall; the thoracically translocated parts of the liver (1); the ventral (2) and the dorsal (3) loop of the left ascending colon; the diaphragm and the area of the dorsal diaphragmatic defect (white arrow); the lung (4) the stomach (5) and the spleen (6).

The foal was subjected to a complete post-mortem examination. In the abdominal cavity, a marked generalized congestion and the presence of 500 mL of serosanguineous fluid were observed. An ovoid, firm, approximately 16 × 12 cm large opening, with no signs of hemorrhage, was located in the right dorsolateral part of the diaphragm (Figure 4a–d). Through this foramen, the viscera from the abdominal cavity, including the small intestine, the cecum, ascending colon, stomach, spleen and a part of the liver (the left and the quadrate lobe) were displaced in the thorax (Figure 4d). No hernial sac was identified, and no broken ribs were observed. In the thorax, the left ascending colon with the pelvic flexure and the jejunal tract were in direct contact with the dorso-caudal parts of the heart and the large vessels (Figure 4d). The right lung was hypoplastic, and appeared to be around a third of its size, whereas the left lung was moderately compressed by the herniated organs. The displaced liver was enlarged and slightly increased in consistency. No morphological changes were observed in other organs. This condition was diagnosed as a congenital diaphragmatic hernia (CDH) with pleuroperitoneal diaphragmatic eventration.

For histology, the samples were fixed in 10% neutral buffered formalin, embedded in paraffin following the routine processing protocol and stained using hematoxylin and eosin (H&E). Histopathology revealed a moderate chronic, passive liver congestion, and in the lung a diffuse congestion with mildly thickened interstitium.

During the following days, the mare’s clinical examination was normal. The ventral abdominal edema had greatly reduced, and a week later, the mare was discharged from the hospital.

## 3. Discussion

In humane medicine, the term “polyhydramnios” is used to describe an increase in the amount of amniotic fluid (>2 L), which is commonly associated with fetal malformations. Central nervous system malformations are the most common cause of hydramnios (up to 45%). Gastrointestinal tract anomalies are the second most common cause, and occur in about 30% of cases. Abnormalities of the cardiovascular system, renal malformations, and other sporadic cases are associated with hydramnios in the remaining 25% of cases [20]. In women, in a study of 76 cases of persistent non-visualization of the stomach by ultrasound exam, in 38 cases (50%) there was an abnormality of fluid volume: 24 were polyhydramnios and 14 oligohydramnios; of these, two cases were congenital diaphragmatic hernia [21]. In the present study, the fetal stomach had never been visualized during ultrasound checks. Even though thoracic and abdominal fetal organs could be identified during transabdominal US evaluation from 6 months throughout the term [14,22], in this case it was not possible, probably due to the ventral edema of the mare and the amount of the amniotic fluid that did not allow visualization of the entire fetal thoracic and abdominal cavity. The most constant aspect was the reduced fetal viability, as indicated by the absence of fetal tone and complex movements with persistent bradycardia detected during every US evaluation. The owner was warned that the US findings were suggestive of a poor fetal outcome, probably due to a supposed congenital defect which was probably the cause of hydramnios.

It is well known that hydrops predisposes to the rupture of the prepubic tendon [9], and in the present case report, the bloody mammary secretion, clinical signs of the mare, and the increase in serum CK activity could strongly support a partial rupture or tearing. The use of an abdominal support bandage was likely enough to prevent total rupture of the prepubic tendon.

Many authors suggest the induction of parturition to deliver the fetus and expel the fetal fluids, because the mare’s survival in these cases is a priority [9,23].

The swelling of the foal, particularly of the head, could be due to an increase in fetal volume from an excessive fluid in subcutaneous tissues. In human cases, the diagnosis of hydrops fetalis is made prenatally and could be due to immune or non-immune mechanisms; the latter include infections, chromosomal abnormalities, and congenital anomalies including pulmonary hypoplasia [24], as occurred in the present case.

Congenital diaphragmatic hernia (CDH) is a rare musculoskeletal defect defined by the presence of an orifice in the tendinous or muscular part of the diaphragm, which causes migration of abdominal viscera into the thorax [25]. Most horses with CDH have a pleuroperitoneal hernia with direct communication between the abdominal and thoracic cavities [26]. Diaphragmatic hernia can be, as well as developmental (CDH), secondary to rib fractures during dystocia (acquired DH) [25,26]. It can be difficult to differentiate CDH and ADH in horses. In general, left dorsal defects are developmental, whereas ventral lesions are parturition-related. Other criteria used include age, gross and histologic appearance of the opening borders, relation of the size of the opening to the volume of displaced abdominal content, absence of diaphragmatic tissue, and coexistence of other developmental anomalies [26].

In the foal in this case, signs of fractured ribs or acute inflammation of the opening were not present; furthermore, the defect of the diaphragm was too small to accommodate the volume of herniated intestine. These findings, associated with the dorsolateral anatomical localization, pointed to a diagnosis of CDH.

Lung hypoplasia associated with congenital diaphragmatic hernias, as seen in our case, is frequently reported in both human and foal cases [25,27,28] of CDH. Although this frequent association suggests that the cause of CDH can be found in primary pulmonary defects, Keijzer et al. [27] formulated a “dual-hit hypothesis” that explains pulmonary hypoplasia by two consecutives developmental insults: initially, a common insult of diaphragmatic primordia which perturbs the organogenesis of both structures, and consequently, a compression caused by the passing of the viscera through the diaphragmatic defect [27]. Therefore, CDH appears to be a primary developmental defect [28].

The generalized congestion that the foal exhibited may be the consequence of persistent pulmonary hypertension caused by congenital pulmonary alterations, which resulted in underdevelopment of the pulmonary vascular system and increased pulmonary vascular resistance.

## 4. Conclusions

Although in this case the mare recovered uneventfully, the diagnosis of hydrops could suggest the presence of fetal anomalies and the need for a very careful transabdominal ultrasound examination. In more severe cases, a complete ultrasound examination could help in choosing whether or not to induce parturition to preserve mare’s life and reproductive career.

## Figures and Tables

**Table 1:** Trend of serum creatine kinase (CK) concentration during the hospitalization period.

Parameter	Day 268	Day 271 *	Day 277	1 Day Post-Partum
CK (U/L)	136	857	107	538

* First day of treatment.

**Table 2:** Foal serum biochemistry and fibrinogen at birth with reference values in brackets [17].

CK (U/L)	538 (40–909)
Total bilirubin (µmol/L)	49.42 (22.23–76.95)
Total proteins (g/L)	41.8 (43–81)
Albumin (g/L)	31.8 (25–36)
Albumin/Globulins	3.18
Urea (mmol/L)	1.28 (0.32–1.43)
Creatinine (µmol /L)	271.4 (106.08–380.12)
Phosphate (mmol/L)	2.13 (1.81 ± 0.58)
Calcium (mmol/L)	4.05 (2.93 ± 0.5)
Sodium (mmol/L)	147 (141 ± 18)
Potassium (mmol/L)	9 (4.6 ± 1.0)
Chloride (mmol/L)	103 (102 ± 12)
Magnesium (mmol/L)	1.10 (0.99 ± 0.74)
SAA (μg/dL)	9 (0–36.600)
Fibrinogen (g/L)	1.59 (1–4)

## Data Availability

Not applicable.
